# Subcutaneous sensors for monitoring congestion and to reduce heart failure hospitalizations—a viable middle ground between deep implantable intravascular monitoring devices and wearable technologies?

**DOI:** 10.1007/s10741-025-10529-8

**Published:** 2025-06-09

**Authors:** Friedrich Wetterling, Bartlomiej Fryc, Ilaria Facchi, Toshimasa Okabe, E Kevin Heist, Marat Fudim

**Affiliations:** 1 Medical Circuits and Systems Laboratory, Discipline of Electronic and Electrical Engineering, Trinity College Dublin, Dublin, Ireland; 2 Trinity Centre for Biomedical Engineering, Trinity College Dublin, Dublin, Ireland; 3 Swedish Heart and Vascular Institute, Seattle, WA USA; 4 Demoulas Center for Cardiac Arrhythmias, Massachusetts General Hospital, Harvard Medical School, Boston, MA USA; 5 Division of Cardiology, Department of Medicine, Duke University Medical Center, Durham, NC USA

**Keywords:** Congestive heart failure, Subcutaneous sensors, Decompensation

## Abstract

Congestive heart failure (CHF) remains a leading cause of hospitalization and mortality worldwide. Continuous monitoring is crucial for early detection of decompensation, potentially reducing hospital admissions and improving outcomes. Cardiac implantable electronic devices (CIEDs) have been established as useful therapeutic interventions that also support continuous monitoring in order to detect early signs of decompensation. However, prior to CIED implantation, effective continuous monitoring solutions are lacking. They exist at two extremes: deep implantable intravascular solutions such as pulmonary artery pressure sensors, which are effective but costly and complex, and wearables, which are inexpensive but lack evidence of their effectiveness and depend on ongoing active patient adherence. Subcutaneous sensors may represent a promising intermediate solution—offering continuous monitoring with lower invasiveness and cost, while maintaining higher adherence compared to wearables. This review explores the role of subcutaneous sensors in CHF management, comparing existing daily trend data to deep implantable sensors measuring direct filling pressure and CIEDs for multi-parametric risk scoring. We discuss their feasibility, limitations, and future integration into routine clinical practice.

## Introduction

Heart failure is a health burden affecting more than 64 million patients globally [1]. Patients hospitalized for congestive heart failure (CHF) are at high risk for readmission and return frequently back to hospital when managing their condition at home [2], leading to worse clinical outcomes and increased healthcare cost. While invasive assessment of congestion uses right heart catheters (RHC) to measure right atrial pressure (RAP), and pulmonary wedge pressure (PCWP) [3], it remains challenging to monitor the progression of congestion remotely [4] and even clinically at follow-ups [5]. The invasiveness of RHC limits practical access to those hemodynamic parameters that are essential for management and therapy [6]. Remote CHF monitoring has been proposed to reduce reoccurring heart failure hospitalization [7, 8]. Monitoring solutions can be categorized into three groups. The first includes cardiac implantable electronic devices (CIEDs), such as pacemakers and implantable cardiac defibrillators. Primarily designed for therapeutic purpose, they now integrate monitoring capabilities such as HeartLogic [9] and OptiVol [10]. Index scores derived from those multi-parametric sensors have been linked to improved risk stratification [11]. However, the application of these devices primarily indicated for patients requiring rhythm management, limiting their broader application to patients who do not have an indication for CIEDs. The second category includes implantable hemodynamic sensors (IHSs)—which are invasive yet small and mostly battery-free devices implanted for instance within the pulmonary artery to directly measure hemodynamic parameters like pulmonary artery pressure (PAP) [12]. While they provide hemodynamic measures, real-time data, and have demonstrated clinical benefits, they are expensive and associated with procedural risks. The last group comprises wearable sensors - devices such as remote dielectric sensors (ReDs) [13] – that provide accessible because non-implantable and low-cost monitoring. However, their efficacy is challenged due to the lack of sensing correlation with internal hemodynamic changes [14], ultimately leading to reduced data quality and limited predictive accuracy [15].

**Fig. 1:**
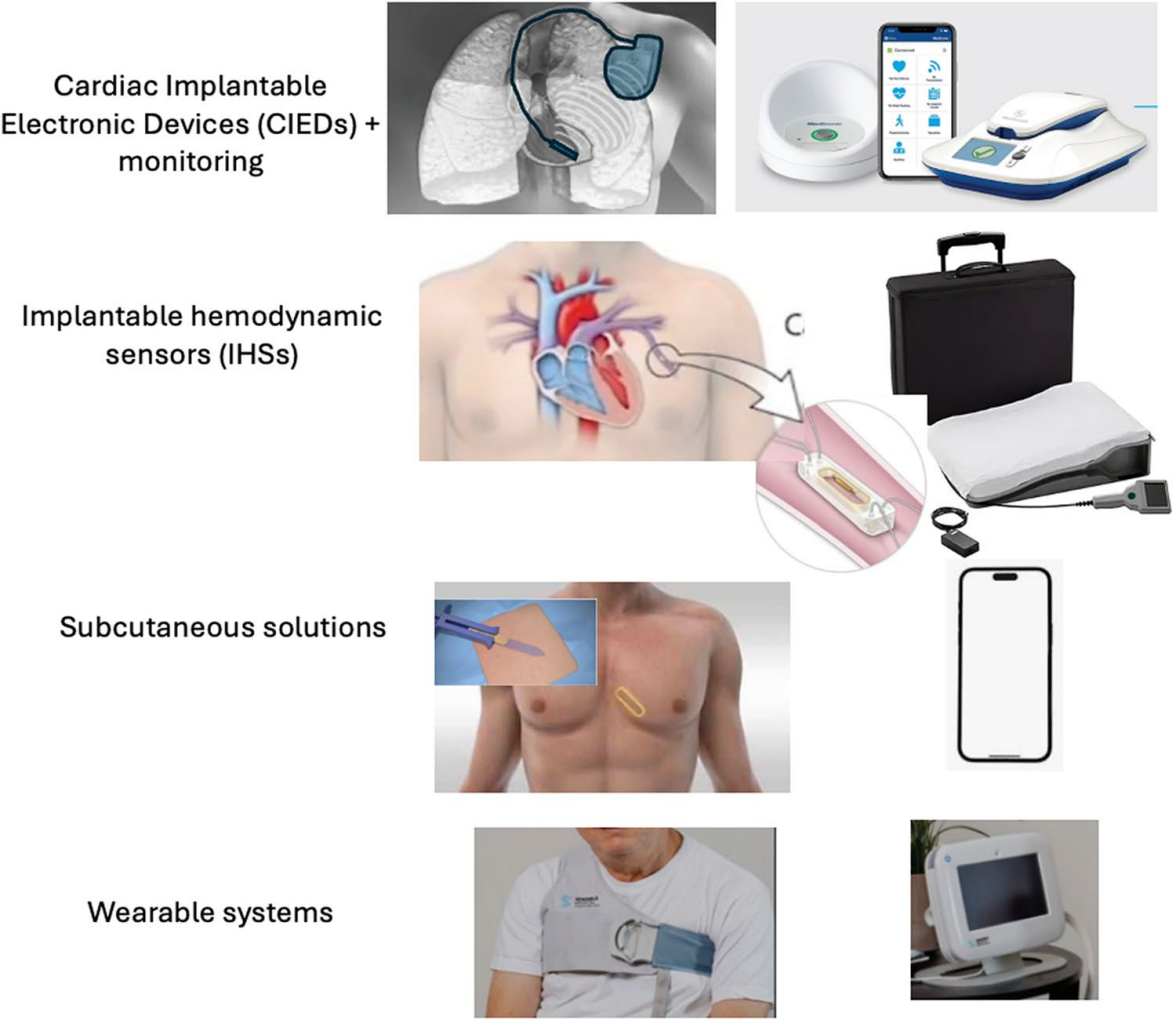
General groups of monitoring options already in place used for therapy cardiac implantable devices (top row), deep implantable intravascular devices (second row, the CardioMEMS sensor) measuring hemodynamic parameters such as cardiac filling pressures, subcutaneous sensors (third row) as less invasive option compared to CIEDs but with similar multi-parametric sensing option, and wearable solutions such as the ReDS (bottom)

Subcutaneous sensors have emerged as a compelling alternative—offering a balance between accuracy, invasiveness, cost, and patient adherence. These devices remain continuously active without requiring daily engagement, potentially providing a more reliable monitoring solution. Subcutaneous implants, such as implantable cardiac monitors (ICMs) or loop recorders, designed to continuously monitor electrocardiography (ECG), have been successfully used for remote atrial fibrillation (AF) monitoring. ICMs are available from BIOTRONIK (Biomonitor) [16], St. Jude Medical (Confirm DM2100, now Assert-IQ by Abbott) [17], Medtronic (LinQ, previously Reveal XT) [18, 19], and Boston Scientific (Lux-Dx Insertable Cardiac Monitor) [20]. With further hardware development, those sensors could well serve the need to monitor CHF patients. A summary of those four sensing approaches is displayed in Fig. 1.

The aim of this review is to explore subcutaneous sensors compared to CIEDs, deep implantable intravascular sensors (i.e. IHSs), and wearable solutions in their capabilities to monitor physiologically relevant trends, as a means to evaluate risk of congestive heart failure events, and to reduce hospitalizations.

## Cardiac implantable electronic devices (CIEDs)

**Fig. 2:**
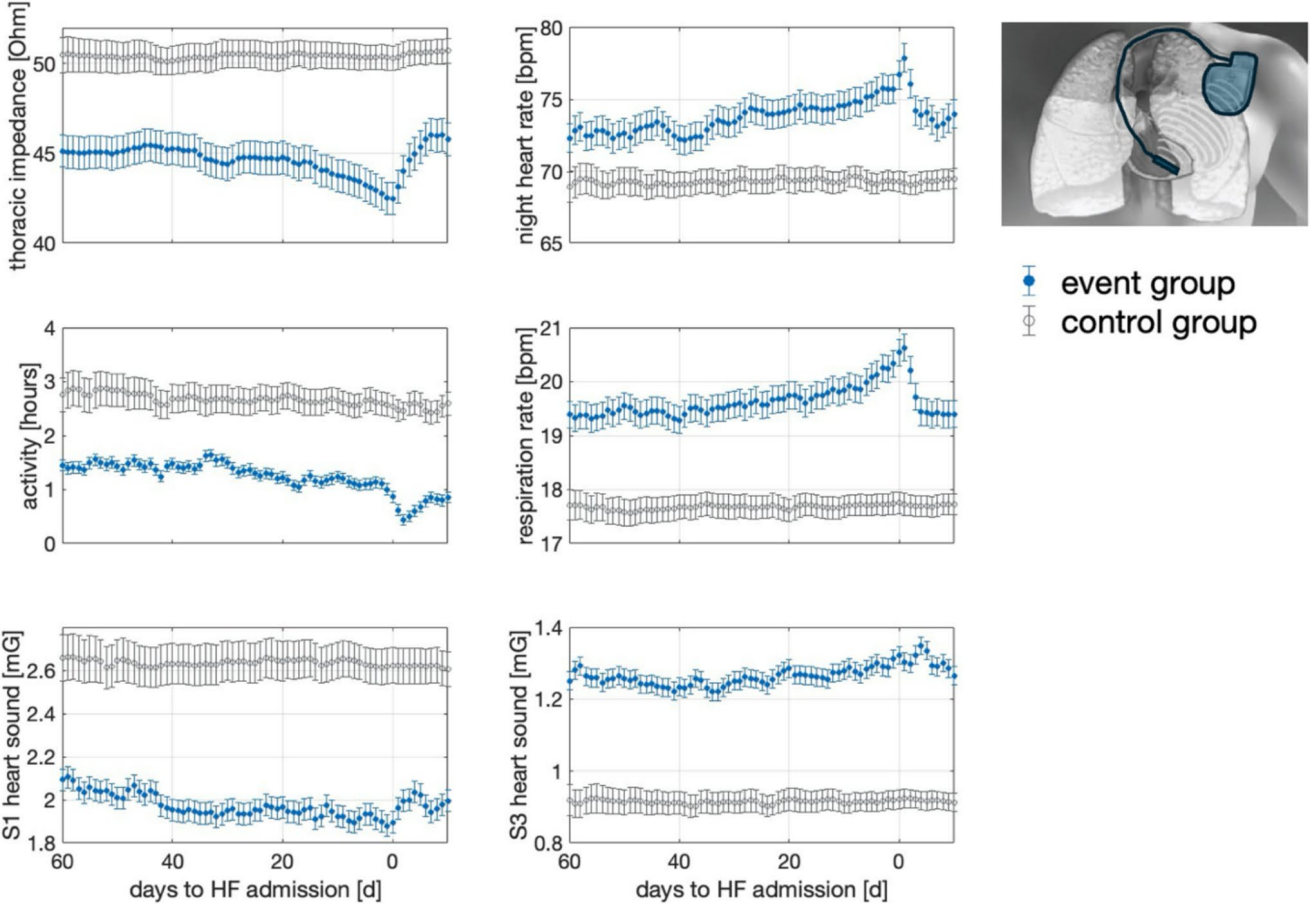
Average trend data for 52 events and 622 non-events offered by CIED and add-on HeartLogic by Boston Scientific (data reproduced from [45]). Note the similarity of those trends with the deep implantable sensor trend in Fig. 2. Particularly impedance has been effective in capturing fluid accumulation using CIEDs

The development of pacemakers and their implementation using subcutaneously implanted pulse generators opened up the opportunity to use existing implant hardware as sensing solutions close to the heart and lungs. Early trials replaced pacemaker leads for pressure sensing leads, as for instance for the Chronicle device [21], and in a larger patient cohort, initial trend analysis showed a slow rise in pulmonary artery pressure (PAP) from an already elevated value in patients who experienced events compared to controls [22]. However, direct measurements of rather low filling pressures remain a challenge with limited means to compensate for atmospheric pressure shifts and drift due to sensor-tissue integration. CIEDs-based monitoring is appealing as it does not require such additional bespoke sensor implantation, but it rather uses established hardware originally dedicated for therapeutic pacing and defibrillating application. The HeartLogic is an example of an algorithm that was developed to predict HF events. It was tested in the MULTISENSE trial where 900 patients were followed up for 1 year. The algorithm provided a median early warning time of 34 days prior to HF events (HFEs) [23]. Figure 2 shows the trend for six features (heart rate, respiration rate, impedance, activity, S1 and S3 heart sounds) recorded by the HeartLogic algorithm in patients that experienced events. The slowly worsening progression of features in the weeks prior the event is apparent. Notably, the features were already significantly different to the features recorded for the non-event group at 60 days prior. This multi-parameter approach shows the power of CIED data to offer trend information that is very similar in profile compared to hemodynamic trends inside the heart. Furthermore, pacemakers and implantable cardiac defibrillators (ICDs) have long-established reimbursement codes, making them widely accessible for CHF patients who qualify for these devices. The integration of CHF risk scores like HeartLogic and OptiVol has enhanced their value further, justifying their cost within the healthcare systems. However, they remain an exclusive option primarily for advanced heart failure patients who require rhythm management.

## Implantable hemodynamic sensors (IHSs)

**Fig. 3:**
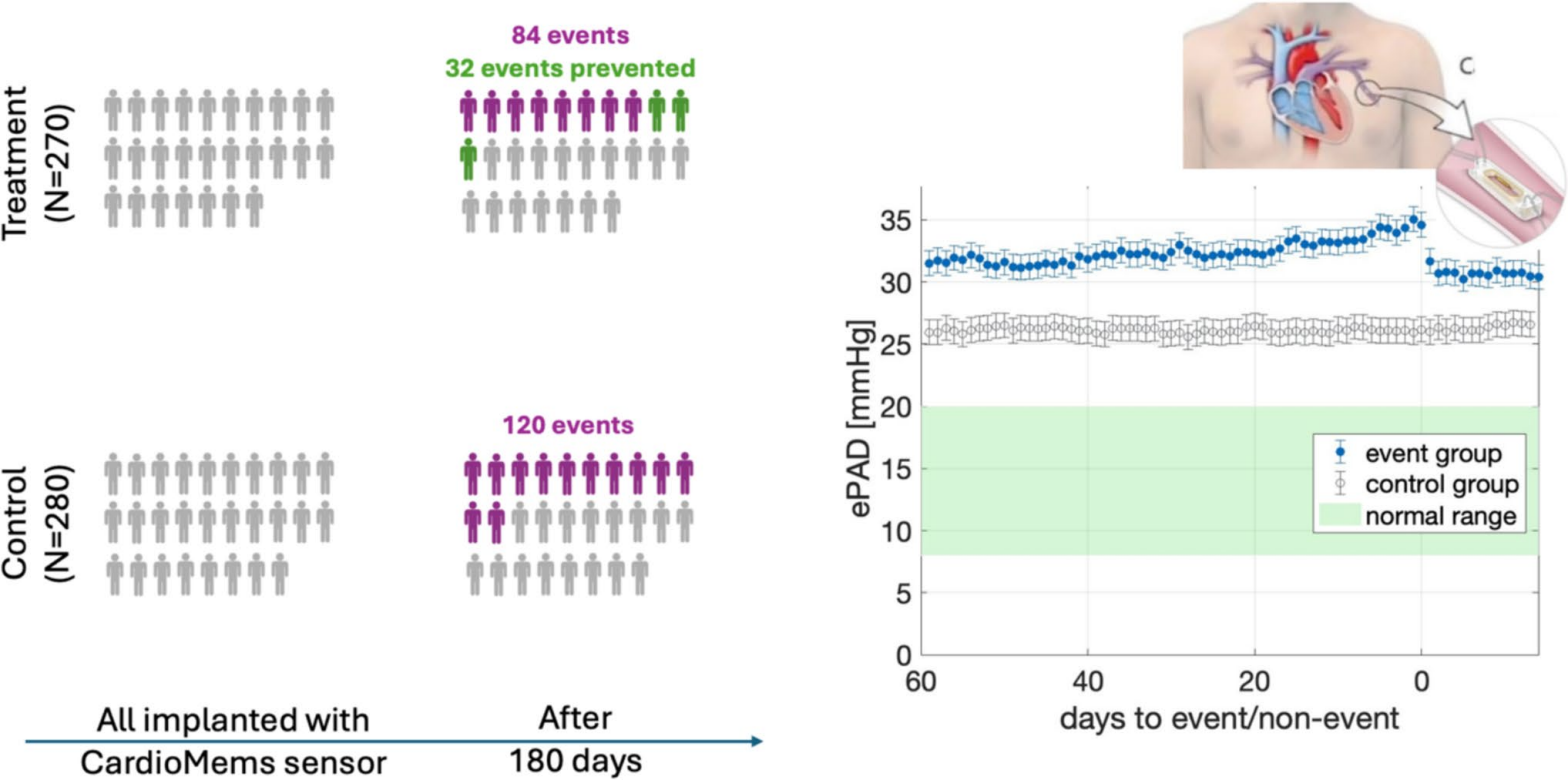
Deep implantable intravascular sensors can record filling pressures directly and have shown benefit in reducing heart failure hospitalization as for instance for CardioMEMS (left, Champion trial results reproduced from [12], each icon represents ten participants, coloured icons represent approximated numbers in multiples of ten) while early intravascular measurements of filling pressure via catheters connected to subcutaneous cans enabled long-term recordings of worsening before events (right, reproduced for recordings taken with Chronicle sensor from [22]). Note that both event and non-event groups ranged well above established threshold with event patients trending up rather marginally in pressure by less than 3 mmHg within 2 weeks prior the event

For patients not indicated for CHF-monitoring CIEDs, deep implantable hemodynamic sensors may offer an alternative to facilitate monitoring. The advent of battery-free small implantable sensors that can be powered and read out via an externally held reader evolved first through the development of the CardioMEMS sensor and system now distributed by Abbott. The sensor measures filling pressure directly, but requires a catheterization laboratory with X-ray capability and a cardiologist with training in transvenous procedures to implant. The CHAMPION-HF trial showed significantly reduced hospitalization rates in patients where physicians used the sensor to adjust treatment [12]. Figure 3 shows graphically that after 6 months, 270 treatment patients had 32 fewer events less compared to the control group. Considering a conservative saving of $20,000 per event, this makes a substantial savings for the health system on top of the improved quality of life for patients. However, it remains unclear whether the cost to implant all 270 patients with this system at a cost of approximately $20,000 per system is cost-effective and whether the benefit justifies the implantation risk, particularly if less invasive solutions which provide similar diagnostic accuracy are available. The adjudicated serious adverse event rate was 3% in the CHAMPION-HF trial [24]. More critically, a randomised control trial comparing PAP-guided HF management versus standard of care failed to show a benefit [25]. Furthermore, these devices are designed to be permanently implanted and are not retrievable. While they carry no battery, they do rely on external powering of the devices through an external reader antenna. From a usability perspective, deep implantable hemodynamic sensors are comparable to wearables. Patients need to actively record data daily during a procedure that may take up to 5 min. The added burden on patients can potentially negatively impact adherence. Other companies are following in the path of CardioMEMS including Endotronix [26], FIRE1 [27], and Vectorius [28]. More advanced CHF patients may benefit from such hemodynamic recordings. To date, it remains unclear how these hemodynamic readings compare to multiparameter-sensing from CIEDs or their alternatives. The use of such deep implantable sensors may become appealing when equivalent outputs to right heart catheterization are provided, that is cardiac output, right atrial pressure, right ventricular pressure, PAP, and PCWP, as well as systemic vascular resistance. Accuracy remains a further issue with numerous reports describing inaccurate measurements for PAP. Indeed, deep implantable sensor may require more frequent recalibration than anticipated to chronically and accurately measure hemodynamic values inside vessels [29].

CHF patients can suffer from prolonged vasoconstriction. Therefore, worsening HF and congestion through intravascular volume accumulation may indeed occur without much or even any pressure increase in the weeks and months prior to hospitalization [30]. However, direct remote measurement of blood volume remains challenging. The most promising IHS innovation originates from a novel implantable sensor in the inferior vena cava (IVC). The FIRE1 first-in-human (FIH) study in 50 patients [31] evaluated the use of an IVC sensor as an implantable version of an ultrasound device to measure IVC geometry remotely returning its own cross-sectional sensor area and collapsibility index without having to implant a battery. Indeed, the results demonstrated a reduction in heart failure hospitalizations (HFHs) at 6 months post-implantation to ~ 20% compared to 100% HFHs prior to implantation (note: prior HFH was an inclusion criteria). Notably, treatment decisions were exclusively guided by clinical consensus rather than sensor readings during the trial. Hence, the post implant HFH rate compared well to similarly low rates of 20% reported for CHF patients in equally well developed countries, such as Sweden [32]. Association of the IVC reading with volume remains vague. To date, IVC readings showed, however, excellent correlation to pressure in a single patient [33]. No direct comparisons or norming of IVC sensor area to blood volume have been presented to date. Existing data suggests thresholding is based on an individualized IVC sensor area and collapsibility index—similar to clinically established guideline-driven approach to predict pressure from IVC diameter [34].

Modest improvements shown by IHSs raise an important question about heart failure management—specifically, whether the field should pursue more advanced invasive, permanent monitoring systems such as for IVC or PAP, or instead focus on developing more effective and accessible outpatient interventions supported by actionable monitoring data. Unsurprisingly, the success of remote monitoring hinges on the availability of actionable and effective non-hospital-based treatment options, which remain limited and may significantly confound current efforts to optimize HF management.

In summary, IHS devices are associated with high costs and complex implantation procedures most appropriate for advanced CHF patients. The required added infrastructure and trained personnel to manage these patients imposes a significant administrative burden on the healthcare system. The lack of broadly funded reimbursement codes by insurances complicates their accessibility, limiting their use to select patient populations covered by specialized programs or clinical trials.

## Subcutaneous sensors

Recent advances by Medtronic to expand the LinQ sensing by adding impedance and activity could potentially enable translation of algorithms developed for CIEDs and congestive heart failure monitoring to subcutaneous devices [35]. Subcutaneous sensors require a minimally invasive implant procedure, come at a lower cost, and have an established reimbursement model. These devices hold the potential to bridge the gap for heart failure diagnosis in patients without a CIED indication and provide a more accessible alternative to deep implantable hemodynamic sensors. The Boston Scientific LUX-Dx [20] and Medtronic Reveal LINQ [18] have established pathways for insurance coverage, particularly in cardiac rhythm monitoring applications. The ALLEVIATE-HF study, an ongoing feasibility study led by Zile et al. [35], is evaluating the expanded use of LinQ with impedance monitoring, which may enhance CHF management. However, impedance monitoring increases power consumption, potentially reducing device longevity. Future Cardia is an emerging company developing an investigational device that is a slightly larger subcutaneous sensor integrating three-axis accelerometer and ECG, aiming for longer battery life and sustained CHF monitoring [36]. These advancements position subcutaneous sensors as a cost-effective alternative that could be reimbursed under existing pathways for insertable cardiac monitors (ICMs) or emerging CHF-specific codes.

While subcutaneous sensors require implantation, this procedure does not require specialized interventional cardiologists, nor does it require lengthy catheter laboratory procedures. Implantation can be as quick as 4 min from skin incision to suture [37], and could be performed in an office setting. Additionally, subcutaneous devices can be easily and safely removed if needed. Data is normally recorded at regular intervals during the day or when triggered by an atrial fibrillation event. Recordings can vary depending on the device but are on the scale of several seconds to a minute per capture. These devices are powered by batteries that enable capturing of low signal ECG and minute acceleration differences. For impedance measurements, the battery can also be used to supply a current in between electrodes. The potential use of other measurements captured by subcutaneous devices is conceivable with the most promising targeting capture of extracellular fluid accumulation via interstitial pressure sensors, promising pre-clinical trend data have been reported recently [38].

Initially larger devices, like the Reveal XT (62 mm × 19 mm × 8 mm), have been reduced in size, as seen with the LinQ (62 mm × 8 mm × 4 mm). This size reduction was primarily driven by reducing sensing exclusively to ECG. However, additional sensing for CHF will naturally increase battery demands, which is why Future Cardia has slightly increased their device size (58 mm × 15 mm × 4 mm) to boost battery capacity.

Subcutaneous devices offer the advantage of continuous monitoring at rest, particularly during nighttime. In contrast, deep implantable sensors and wearable devices require patients to actively take recordings at a fixed time during a day, which may not capture a true resting state. This improves patient compliance and may help reduce hospitalizations.

**Fig. 4:**
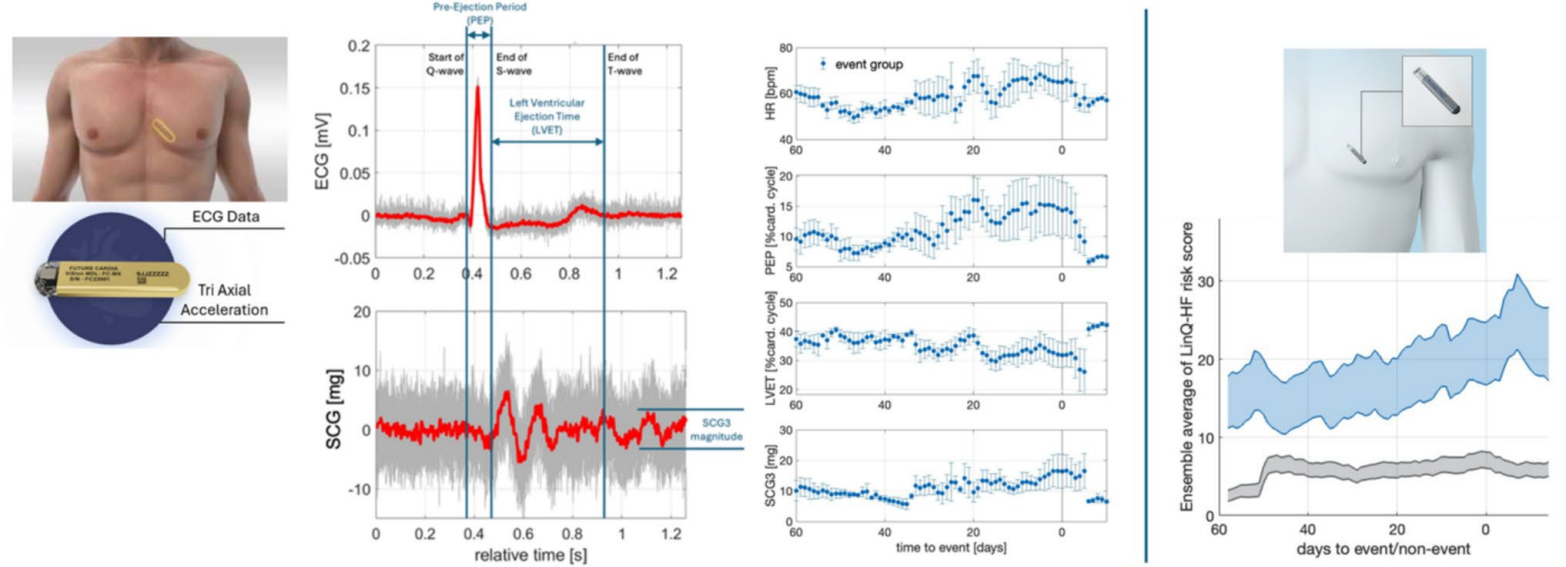
Example cardiac cycle data captured and extracted feature trend data from subcutaneous device by FUTURE CARDIA for three patients and four events (left of blue vertical line, presented by M. Fudim at THT, Boston, 2025). HR, heart rate; LVET, left ventricular ejection period; PEP, pre-ejection period; SCG, Seismocardiography. An example trend of how similar features can be summarised in a single number risk score is shown on the right for the LinQ device extracted for 144 patients and 38 events (blue) and for non-event patients (grey) (reproduced from [35])

In summary, subcutaneous devices allow HF physicians to implant and manage their patients’ data and treatment more effectively. This could position them as the next generation of implanters, giving them greater control in managing their patients. Trend data from the Future Cardia device for three patients that experienced four events can be seen in Fig. 4. An example of how a risk score trends for a similar system is shown in the same figure based on data published for a larger study in 144 patients using the LinQ. First result from the ALLEVIATE-HF trial for 59 enrolled patients showed that a personalized medication intervention-based ICM risk score can be safely instituted in patients with HF, irrespective of symptoms [39]. Future studies need to compare subcutaneous sensors to deep implantable sensors (CardioMEMS) and CIED-based monitoring (HeartLogic, OptiVol) to compare clinical outcomes.

## Wearable devices

Similar to CIEDs, wearable solutions such as ReDS [13] have the advantage of providing monitoring without an additional implant procedure. While results for ReDS-SAFE-HF trial reported less events experienced in the guided study group, the overall numbers of events was low and the exact management protocol remains unclear [40]. No trend data is available for this system hampering a further evaluation of its use in individual cases. Naturally, the external application of sensors is challenging, particularly for elderly patients and patients that suffer from debilitating illness. Gripping devices itself may be a challenge to some patients, and positioning of sensors day to day hampers regular and reliable data capture. Most notably, the sensing is patient driven and requires patients to capture data on the scale of several seconds to minutes daily. Those devices require more effort from patients on top of having their base station powered and connected to the internet. To date, no trend data has been identified that shows similar trend evolution as has been shown for CIEDs, deep implantable intravascular sensors, and subcutaneous sensors. A summary of existing options and their respective key features is listed in Table 1.

**Table 1:** Summary of general monitoring options for congestive heart failure management

Monitoring solution	Invasiveness type and necessary procedures	Output	Comment
CIEDs + monitoring logic	Hardware in place already as treatment (pacemaker) or rescue (defibrillator) options	Heart Failure Related Evente (HFRE) risk score from multiple sensor features (impedance and acceleration sensing based while ECG is used for treatment triggers)	Limited extra cost for external units
Implantable hemodynamic sensors	Deep implantable small sensor in intravascular space requiring additional transcatheter procedure in X-ray providing catheter labs and cardiac interventional specialist	Provide pulmonary artery pressures (CardioMEMS, Endotronix) or LAP (Vectorius) as calibrated against right heart catheter systems	High cost for implantation of non-retractable sensors, reimbursement uncertain
Subcutaneous sensors	Minor office-based procedure	HFRE risk score from multiple sensor features (impedance and acceleration sensing based while ECG is used for treatment triggers)	Retractable, reimbursement existing for ECG monitoring, added new features from acceleration may require further qualification, existing devices in market (510 k possible)
Wearables	Non-invasive, requires patient to capture readings	Risk score based on lung water estimation via radiofrequency	Affordable, yet efficacy is lacking

## Clinical evidence

Assessing clinical efficacy for early detection systems in CHF patients presents inherent challenges. The ultimate goal is twofold: first, to detect an impending HF event, and second, to trigger an effective intervention that can prevent the event from occurring. Thus, the effectiveness of the system depends not only on accurate detection, but also on timely clinical action and appropriate as well as effective therapeutic response.

In this review, the sensor trend data itself was evaluated primarily on its ability to identify physiological changes associated with heart failure decompensation, with clinical events adjudicated independently. However, as in other studies evaluating heart failure monitoring technologies of IHSs, clinical endpoints such as reduction in HF hospitalizations are typically reported rather than direct measures of detection efficacy alone. This reflects a broader challenge: the detection of risk alone does not equate to prevention unless followed by successful clinical intervention. As such, while sensitivity and specificity are important indicators of system performance, they are only part of the overall effectiveness of a monitoring strategy.

CIEDs showed signs of clinical evidence in various clinical trials. For instance, the IN-TIME Trial demonstrated improved clinical outcomes, including reduced mortality, through multiparameter remote monitoring [41]. Furthermore, the MultiSENSE Study (HeartLogic) reported sensitivity of ~ 70% with a median early warning time of 34 days prior to HF events [42]. Other in depth reviews deliver more detailed reports of area under the curve, sensitivity, specificity, and false alert rates from validation studies [43]. Those particularly emphasize the predictive window and integration of parameters (e.g. thoracic impedance, heart rate variability, activity). Hence, CIED risk scores support clinical actionability—the capacity for clinical teams to intervene based on an indexed threshold crossing.

IHSs on the other hand are more vague on defining actionable thresholds, yet reported significant reductions in overall hospitalizations. For instance, CHAMPION-HF demonstrated a 33% reduction in HF hospitalizations over 15 months with pulmonary artery pressure-guided therapy [44]. GUIDE-HF presented, however, mixed results with significant impact in pre-pandemic subgroup analyses [25]. Notably, they report hospitalization reduction rates, yet depend strongly on detailed management protocols and specialized clinician engagement.

Comparing both approaches, IHSs offer precise pressure trends of mostly a single clinically relevant parameter, while CIED indices offer a broader physiologic picture of a complex syndrome. CIED alerts are automated within existing workflows and IHS data requires deliberate daily review and clinician directed adjustment as thresholds are either undefined or highly individualised.

In summary, CIED-based risk indices and IHSs each demonstrate robust efficacy in reducing HF events, though through differing mechanisms. Multiparameter risk indices offer validated predictive capability with broader scalability and ease of integration, while hemodynamic sensors demonstrate potent event reduction in select, intensively managed cohorts. Optimal strategy selection may hinge on patient characteristics, healthcare setting, and resource availability.

CIED-based risk indices offer a broader, validated, scalable, and safer approach to predicting and preventing HF hospitalizations, leveraging multiparametric diagnostics with proven sensitivity, specificity, and acceptable false alert rates. Although IHSs demonstrated significant benefit in highly controlled settings, the integration ease, patient safety, and cost-effectiveness favour the risk index strategy—especially when generalized to a wider HF population with pre-existing CIEDs.

## Future directions and clinical integration

There is a potential for expanding the existing indications for subcutaneous sensors from AF monitoring to CHF monitoring. Subcutaneous sensors have already demonstrated value in arrhythmia detection and early CHF decompensation monitoring. However, further advancements could expand their role in personalized heart failure management. Artificial intelligence (AI)–driven analytics could optimize individualized alerts, reducing false positives and improving early intervention strategies. Multi-parameter monitoring through integration of ECG, respiration rate, activity levels, and added sensor metrics could enhance predictive accuracy for CHF events. In combination with wearable technology, hybrid models that combine subcutaneous monitoring with patient-friendly wearables like smart watches could improve overall disease management while maintaining convenience.

Clinical trials are needed to evaluate the efficacy of subcutaneous sensors for CHF monitoring. To gain a broader clinical adoption and reimbursement, subcutaneous sensors must demonstrate clear clinical benefits in large-scale studies. Examples of such studies include evaluation of CIEDs in the MultiSENSE trial for 900 patients [23] and for deep implantable sensors in the CHAMPION-HF for 550 patients [44] and GUIDE-HF for 1022 patients [25].

With the growing demand for minimally invasive and cost-effective CHF monitoring, regulatory agencies and healthcare insurers may begin supporting subcutaneous sensors as a mainstream CHF management tool. Together with enhanced resources and hospital system infrastructure, those innovations must be designed to handle the administrative burden of remote monitoring data sources in the day-to-day clinical workflow. Overall, this shift could lead to more favourable reimbursement pathways similar to those for ICMs. Furthermore, it may offer new innovative solutions for integration with telemedicine platforms, allowing real-time physician alerts for CHF deterioration. Both will ultimately lead to greater adoption by heart failure clinics, reducing reliance on expensive and highly invasive options.

## Conclusion

Subcutaneous sensors represent a transformative technology for CHF monitoring—offering a compromise between invasive deep implantable sensors and wearables. Their continuous, passive monitoring ensures high patient adherence, while their lower cost and minimal invasiveness make them a viable alternative to both CIED-based and deep implantable monitoring solutions. With emerging reimbursement models, ongoing feasibility studies, and technological advancements, subcutaneous sensors are well-positioned to become an integral part of CHF management. If they are proven effective for reducing hospitalizations and improving early detection of decompensation, these devices could significantly enhance patient outcomes and reduce healthcare costs, bridging the gap between invasive and wearable monitoring solutions.

## Data Availability

No datasets were generated or analysed during the current study.
